# Issues to Consider When Measuring and Applying Socioeconomic Position Quantitatively in Immigrant Health Research

**DOI:** 10.3390/ijerph10126354

**Published:** 2013-11-27

**Authors:** Signe Smith Nielsen, Nana Folmann Hempler, Allan Krasnik

**Affiliations:** 1 Danish Research Centre for Migration, Ethnicity and Health, Section for Health Services Research, Department of Public Health, University of Copenhagen, Øster Farimagsgade 5A, 1014 Copenhagen K, Denmark; E-Mail: alk@sund.ku.dk; 2 Steno Diabetes Center, Steno Health Promotion Center, Niels Steensensvej 8, 2820 Gentofte, Denmark; E-Mail: nfhr@steno.dk

**Keywords:** migrant, ethnic minority, socioeconomic position, socioeconomic status, health, measurement, quantitative, causal mechanisms, inequality, inequity

## Abstract

The relationship between migration and health is complex, yet, immigrant-related inequalities in health are largely influenced by socioeconomic position. Drawing upon previous findings, this paper discusses issues to consider when measuring and applying socioeconomic position in quantitative immigrant health research. When measuring socioeconomic position, it is important to be aware of four aspects: (1) there is a lack of clarity about how socioeconomic position should be measured; (2) different types of socioeconomic position may be relevant to immigrants compared with the native-born population; (3) choices of measures of socioeconomic position in quantitative analyses often rely on data availability; and (4) different measures of socioeconomic position have different effects in population groups. Therefore, caution should be used in the collection, presentation, analyses, and interpretation of data and researchers need to display their proposed conceptual models and data limitations as well as apply different approaches for analyses.

## 1. Introduction

Migration has been increasing worldwide facilitated by improved transportation and communication techniques [1], and in 2010, migrants were estimated to number 214 million worldwide (3.1% of the world population) [2]. Immigrants constitute a heterogeneous group with respect to their ethnic features, historical roots, religion, culture, values, notions, migration history, and practices concerning health. In some countries, immigrants seem to enjoy a better health than the native-born at their time of arrival to the host country [3,4,5]. This has been ascribed to the “healthy immigrant effect” that healthier persons migrate but also to a strict immigration policy [4]. Yet, in many countries, compared with the native-born, immigrants are often disadvantaged regarding health [4,6,7,8,9]. Additionally, immigrants constitute a potentially socially disadvantaged group with lower educational levels, lower employment rates, lower income, lower wealth, and residence in more deprived neighbourhoods than native-born [10,11,12,13,14]. 

Given the well-known association between socioeconomic position and health [5,15,16,17,18,19] and the fact that immigrants often have lower socioeconomic positions than native-born [10,11,12,20] immigrant-related inequalities in health are largely influenced by socioeconomic factors [5,12,21,22,23]. Therefore, socioeconomic position needs to be taken into account when investigating immigrants’ health in order to understand their health profiles and trajectories. Furthermore this may shed light on the specific impact of other factors such as migration history, genetic differences, and cultural differences on health [23,24,25]. This knowledge can be used as a base for developing health policies that can respond effectively to immigrant-related inequalities in health [12]. Nevertheless, despite the fact that socioeconomic position has been much debated in the scientific literature it has still been used ambiguously in quantitative immigrant health research. To advance our understanding of the relationship between socioeconomic position and health in different population groups, and ultimately foster appropriate policies and practices to improve population health, a more nuanced approach is required. Drawing upon previous findings, this paper discusses issues to consider when: (1) measuring socioeconomic position in immigrants; and (2) “applying” socioeconomic position in quantitative immigrant health research.

## 2. How to Measure Socioeconomic Position in Immigrants

### 2.1. What Is Socioeconomic Position?

Socioeconomic position refers to the social and economic factors of an individual that determine the individual’s social standing within society [26,27]. Socioeconomic position is derived from a particular social context implying that classifications of socioeconomic position will vary in societies with different economic or social structures and across time [26]. The three classical indicators of socioeconomic position include educational level, occupation, and income which represent different pathways through which socioeconomic position can affect health. *Educational level* attempts to capture the knowledge assets of an individual. The effect of educational level on health has been hypothesized as resulting from one or more of the following pathways: (1) more educated individuals obtain less hazardous and higher paying jobs; (2) more educated individuals are more likely to pick up health messages and to avoid health risk and engage in preventive behaviours; (3) more education may also lead to more resilient social psychological status, including sense of control [28,29]. Strengths of the indicator include it is easy to measure, it is stable across life time, it is relevant to individuals regardless of age and working circumstances, and it normally ensures a high response rate. As education is normally completed in early adulthood and in part determined by parental characteristics it may somewhat measure life course socioeconomic position. Limitations include educational level varies for different birth cohorts, it does not obtain information on the quality of the education, and education obtained abroad may be difficult to classify and compare across groups [28]. *Income* reflects material resources which primarily influence health as buying access to better material resources and services as well as fostering self-esteem. Strengths of the indicator include its reflection of material living standards. Limitations include that it can change on a short basis, it is not stable over time (especially for students and retired individuals, it is an unreliable indicator), and it is a sensitive question leading to greater non-response than other socioeconomic indicators [28]. *Occupation* reflects an individual’s place in society and is strongly related to income (and thus material resources). It mirrors workplace conditions and hazards as well as psychosocial processes such as sense of control and autonomy. Strengths of the indicator include its availability, also in routine data, and that it includes a measure of psychosocial processes. Limitations include that it cannot be assigned to individuals outside the labour force (including students; retired persons; and persons whose work is inside the home, mostly affecting women), individuals who are self-employed may be difficult to classify, and the meaning of occupation may vary for different birth cohorts and for different geographical contexts [28]. 

The above mentioned different strengths and weaknesses of the indicators are not universal but will vary with context. Additionally, contexts in relation to culture, norms, history, geography, economy, political landscape etc. in society and over time play a significant role in availability of indicators and on the choice of indicators of socioeconomic position in a given research project. For example, occupation data may be readily available in some European contexts, but is less readily assessed and available in the U.S. Other indicators such as wealth and home ownership are also often used. In some countries, gender, sexual orientation, ethnicity, or religion plays a significant part in what social position an individual hold [26] and may therefore be crucial to include to obtain a more relevant measure of socioeconomic position. The indicators of socioeconomic position may be objective or subjective and can be used either separately, in combination or in indexes. Indicators may also be collected on an individual level, household level or community level (composite indicator, indices of area deprivation) [28]. Despite that individual indicators are often preferred, area-based indicators can also have an independent influence on health [30]. It is important to underscore that the individual indicators of socioeconomic position are still determined by structural relations between groups in society [19]. Each of these mentioned indicators measure different, but often related aspects of socioeconomic position. Indicators of socioeconomic position have been discussed in greater detail by Galobardes *et al*. [19,28,30]. 

### 2.2. How Should Socioeconomic Position Be Measured?

In the discussion of influences of socioeconomic position in the relationship between migration and health, it is important to note that differences in socioeconomic position between immigrants and the native-born population do not fully explain the differences in health [6,7,8,23], also after taking into account genetic factors, cultural factors, and migration history. There are at least four aspects that should be considered: (1) Lack of clarity of measurement of socioeconomic position; (2) the relevance of different types of socioeconomic position differ across population groups; (3) data availability; and (4) differential effects. These aspects are discussed in below. 

#### 2.2.1. Lack of Clarity of the Measurement of Socioeconomic Position

First, there is a lack of clarity about how socioeconomic position should be measured; this is underscored in immigrant research. In order to compare the health status of different population groups and the role of socioeconomic position, the socioeconomic position indicator should measure the same construct across population groups and have the same meaning across time. Nevertheless, there are numerous interconnected pathways whereby the individual’s health is harmed or promoted by the cumulative effects of his or her standard of living, workplace conditions, and social networks at different times in the life course [27,31]. This may also affect different population groups differently and in different times [27,31]. Socioeconomic position has consequences for the individual’s health both before and after migration, and migration as a life change can be a risk factor in itself. Socioeconomic and health differences are likely to be a consequence of a lifetime accumulation of disadvantage among immigrants, including risk and protecting factors in both the country of birth and in the host country, as well as critical development periods and age at migration [19,32]. To better conceptualize, analyze, and interpret immigrants’ health, Spallek *et al*. have suggested a theoretical framework for immigrants’ health in a life course approach which enables the consideration of risk factors, including socioeconomic risk factors, and disease outcomes over the different life phases of migrants [33]. 

#### 2.2.2. Relevance of Different Types of Socioeconomic Position Differ across Population Groups

Second, as Stronks and Kunst have pointed out, different types of socioeconomic position may be relevant to immigrants compared with the majority population [12]. To illustrate this, immigrants may be disproportionally affected by lack of education as, for example, it impedes the ability to learn the language of the host country, leading to poorer jobs and lower income [12]. Another bias in using education as an indicator of socioeconomic position across population groups is the potential lack of comparability of different education systems, including quality of educational experiences [8,28,32]. Likewise, the issue of overqualification may be relevant, e.g., that education level may be much less indicative of socioeconomic position in the host country for immigrants as they are more likely to be underemployed according to their educational level [6,34]. Similar biases are at play for other socioeconomic indicators such as income and occupational class as they may not fully reflect the (dis)advantage in material living conditions among immigrants. For instance, when measuring (disposable) income for an individual, immigrants’ income may be more likely to change and is dependent on length of stay in the host country [32]. Remittances are often not taken into account resulting in biased information of disposable income for immigrants. Furthermore, the socioeconomic indicators do seldom cover other forms of disadvantages, for instance that immigrants are often living in socially and economically deprived neighbourhoods that had an adverse effect on health [14,35]. Despite the well-documented social gradient in health, American studies have generally found a weak or flat social gradient in health among immigrants [32]. Different explanations have been suggested, including the “healthy immigrant effect” and that the imported social gradient from the home country may be in favour of health [32]. Finally, it is worth mentioning the paradox in measuring socioeconomic position in relation to health in immigrants that while income and wealth normally increase with duration of stay in the host country, health decreases [3,4,36]. These findings emphasize that future studies should collect and test different indicators of socioeconomic position, preferably using a life course approach.

#### 2.2.3. Data Availability

Third, choices of measures of socioeconomic position in quantitative analyses rely on data availability. Yet, we often have limited data on socioeconomic position which may result in residual confounding and persistent inequalities might reflect unmeasured aspects of socioeconomic position [31].

#### 2.2.4. Differential Effects

Fourth, different measures of socioeconomic position have different effects in population groups [12,31,37]. There is evidence that ethnic minority individuals do not experience the same returns as the majority individuals for higher socioeconomic position achievements. This is called the “diminishing returns hypothesis” [20,31]. For example, within occupational groups, Whites have higher incomes than Blacks; within income strata, Blacks have lower wealth levels than Whites [23]; poor Black and Latino families are more likely to live in deprived neighborhoods than poor White families [38]; and Blacks do not enjoy similar returns in self-rated health as do Whites with increased income and occupational prestige [20]. Non-equivalence which refers to the argument that socioeconomic position does not have a uniform effect on all population groups is also important. Socioeconomic position may have different meanings to different population and ethnic groups with regards to health outcomes. The origin and processes of social stratification may vary in different population and ethnic groups and consequently the same indicator of socioeconomic position may not capture the socioeconomic distribution in the groups. 

Similarly, the effects of socioeconomic position on health among immigrant men and women have shown to differ. In similar socioeconomic position, immigrant women were more likely than men to have a poor health and to have a low income when employed [39]. Different explanations include that family migration is often in favour of man’s employment [39] and there are different cultural contexts which has impact on gender roles. Wider gender inequalities in immigrants’ countries of origin might exist where women have fewer options and less power which may again be transferred to the family sphere in the host country [39]. Likewise, hazards on health by socioeconomic position are not uniformly distributed among immigrant men and women (which is often also the case among native-born men and women). The cumulative exposures of being in low social position, being an immigrant, and being a women, etc. may place immigrant women in a particular disadvantaged position [32,39]. The fact that socioeconomic position has different meanings for immigrant men and women stresses the importance of testing for interaction effects between socioeconomic position and sex. 

### 2.3. Choice of Socioeconomic Indicator Depends on the Specific Health Outcome

There is no single best indicator of socioeconomic position, as each indicator measures different but often related aspects of socioeconomic stratification and might be more or less relevant to different health outcomes and at different stages in the course of life [19,28]. Relevant measurement of socioeconomic position is dependent on the theories/hypotheses behind—which socioeconomic elements the researchers believe play a role in the concrete association as well as on the concrete data possibilities which again varies across countries and population groups. Therefore, the choice of indicators should be contingent upon the research questions and the proposed mechanisms between socioeconomic position and the health outcome [28]. It is important that the researchers lay out the premises of the data and the hypotheses. An example of such work is the study by Hempler *et al*. [7] who examined whether an effect modification between different indicators of socioeconomic position and country of birth existed, or whether socioeconomic position indicators were mediators of the effect of country of birth on the incidence of cardiovascular disease (CVD) and acute myocardial infarction (AMI). The measurements of socioeconomic position were based on individual registry data, included three different indicators (income from earning and social transfers by tax authorizes used as a continuous variable, employment, and home ownership) and based on data from the preceding year prior to follow-up [7]. Studying health outcomes (CVD and AMI) occurring mostly among the elderly, income and employment might be inadequate indicators in this population group, and home ownership reflecting material wealth throughout the life course might be more valuable. Nevertheless, due to small sample size, the broad categories of socioeconomic position left a possibility of biased results and also the omission of educational data, due to poor validity, was a limitation. Another example of a measure that worked well is the study by Reijneveld *et al*. [8] who examined whether an adverse health status of immigrants could be explained by their socioeconomic position. Socioeconomic position was based on self-reports and measured by three traditional indicators: educational level, occupational status, and income representing different pathways through which socioeconomic position can affect health. Of all socioeconomic position measures, adjustment for educational level yielded the largest reductions in the size of population group differences. The authors ascribed this to the possibility that higher educational level improve the potential of immigrants to integrate in the host country and to benefit from health care [8]. 

## 3. How to “Apply” Socioeconomic Position Quantitatively in Immigrant Health Research

The relationship between migration and health and the role of socioeconomic position is complex. Not only is the relations socioeconomic position complex (as discussed above) but this is also the case for the relationship between immigrants and health. Evidence remains lacking on how the mechanisms of migration influence health [6,12,23,40,41]. Exposure to health risks in immigrants’ home countries, strains in the migration process together with poor socioeconomic conditions, loss of social status, and change of roles may result in a negative stress response and risk behavior, which makes immigrants a particularly vulnerable group in society with special health problems [25,42]. Consequently, there are many factors within a life course perspective to take into account for fully understanding the relationship between immigrants and health [33]. Moreover, data availability of many of these factors is often limited. Acevedo-Garcia *et al*. have suggested the creation of comparable national health surveys in countries of birth and host countries as a first step to further our understanding of immigrant health over a life course [32]. 

Often it is hypothesized that low socioeconomic position might lead to poorer health, but reverse associations between socioeconomic position and health are possible as poor health might lead to unemployment and subsequent lower income. Risk of reverse causality is likely, particularly in cross-sectional studies of immigrant health and socioeconomic position. 

### 3.1. Confounder, Mediator or Effect Modifier

The role of indicators of socioeconomic position may be understood, analyzed and interpreted as a confounder, mediator or effect modifier. Due to the explanatory nature of socioeconomic position on immigrant health differences, it has been recommended that researchers should make every effort to adjust for all conceptually relevant measures of socioeconomic position when comparing population groups [31,41,43]. Therefore, to avoid spurious relationships, most health studies among immigrants that consider socioeconomic position treat this as a potential confounder of the relationship between the exposure and outcome [31]. Accordingly, socioeconomic position, included in quantitative analyses, is often used by migrant health researchers to ‘control’ for, rather than study, the effects of socioeconomic position on health [27,37]. As Nazroo has argued, this leads to two related problems when making comparisons across population groups. First, if socioeconomic position is treated as a confounder with the purpose of uncovering the “real” relationship between migration and health, the results will be presented accordingly. Thereby, the influence of socioeconomic position will be hidden and their effect on immigrants’ health will be lost. Second, if only results are presented after the standardization of socioeconomic position have been applied, differences between immigrants and native-born that are left will be wrongly assumed by the reader to be caused by a cultural or genetic immigrant effect [23]. 

Some studies have demonstrated that migration background has a significant influence on a person’s socioeconomic position [10,12,20] which then affects health [15,16,17,18], and it can therefore be argued that indicators of socioeconomic position should be understood as intermediate variables in this context [31,43]. Other studies have suggested an effect modification between socioeconomic position and country of birth, in relation to health, because the relationship between the indicator(s) of socioeconomic position and the health outcome measure differs between population groups [6,20]. In this light, we suggest a more careful consideration of effect modification and mediation of socioeconomic position on health among immigrants versus native-born. The researcher(s) should make careful considerations of the mechanisms of the relationship between migration, socioeconomic position, and health and demonstrate the conceptual/analytical model as well as the results by the stepwise model and discuss the limitations that apply. 

### 3.2. Applying Socioeconomic Position in Statistic Modelling

When socioeconomic position is conceptualized as a mediator, the statistical methods to assess such a role are mediation tests and when socioeconomic position is conceptualized as an effect modifier, the statistical methods to assess such a role are interaction tests. 

Although it is widely recognized that different measures of socioeconomic position have different effects in different population groups [31], much of the literature on immigrant-related/ethnic differences in health does not use the interaction approach but include only the main effects of country of origin/ethnicity and socioeconomic position [20]. In studies where interactions between country of origin and the different measures of socioeconomic position have been taken into account, several of the interactions have been shown to be statistically significant [6]. This is a strong indication that the effect of socioeconomic position varies across immigrant groups and the native-born population. However, analysis of interactions should be interpreted with caution, as the product term depends on the statistical model. For instance in logistic regression and cox regression models, statistical interaction is implicitly exponential and therefore multiplicative. This means that the researcher might conclude lack of evidence of an interaction between two factors in such models, but might overlook potential interactions that refer to a deviation from additivity. If the researcher is interested in studying whether the combined effect of low socioeconomic position and immigrant status is higher or lower than the addition of both effects in relation to a certain health outcome, a method outlined by Andersson *et al*. can be applied [44]. 

Newer and more advanced methods such as multilevel studies (spatial and longitudinal) and structural equation models can advance our understanding of the complex association between migration and health. Multilevel analyses can be applied in order to determine the effect that area socio-economic circumstances have on a health outcome beyond individual socioeconomic position in studies of population differences in health outcomes. As shown in both a European and American context, analyses incorporating both individual and neighbourhood-level contextual factors showed that adding contextual factors as explanatory variable seemed to attenuate the difference in health between the population groups [14,45]. Structural equation models may be used to identify significant relationships between migration, socioeconomic position and health. Structural equation modeling is a collection of statistical techniques that allow a set of relationships between one or more independent variables, and one or more dependent variables, to be examined. Such models are seen in studies focusing on social support and immigrant health [46,47,48]. There is a slowly growing evidence-base from studies of immigrant health and socioeconomic position applying these methods (e.g., studies of ethnic enclaves of immigrants [49]).

## 4. Conclusions

This paper reflects the ongoing discussion of socioeconomic position within the field of immigrant health research. Immigrant health is especially sensitive to weaknesses regarding dealing with socioeconomic position quantitatively due to the complex methodological issues. Consequently, the research field becomes a lens of these varied and diverse theories, mechanisms and hypotheses which illustrate the different complementary factors at play under varied contexts. When measuring and applying socioeconomic position in immigrant health research, it is important to be aware of four aspects: (1) there is a lack of clarity about how socioeconomic position should be measured; (2) different types of socioeconomic position may be relevant to immigrants compared with the native-born population; (3) choices of measures of socioeconomic position in quantitative analyses often rely on data availability; and (4) different measures of socioeconomic position have different effects in population groups. The choice of indicators of socioeconomic position should be contingent upon the research questions and the proposed mechanisms between socioeconomic position and the health outcome. Caution should be used in collection, presentation, analyses, and interpretation of data and researchers need to display their proposed conceptual models and data limitations. We suggest that future studies should collect and test different indicators of socioeconomic position, preferably using a life course approach, be clear on choice of measurement and the possible limitations and issues concerning validity of the indicators used. Furthermore, we suggest that researchers consider and possibly apply different approaches for analyses, such as the mediation model and the interaction model. More explicit theories and advanced methods should be used to enhance our understanding of the complex association between migration, socioeconomic position, and health.
